# Effects of spatial scale of sampling on food web structure

**DOI:** 10.1002/ece3.1640

**Published:** 2015-08-18

**Authors:** Spencer A Wood, Roly Russell, Dieta Hanson, Richard J Williams, Jennifer A Dunne

**Affiliations:** 1 School for Environmental and Forest Sciences, University of WashingtonSeattle, Washington; 2 Woods Institute for the Environment, Stanford UniversityStanford, California; 3 Sandhill InstituteGrand Forks, British Columbia, Canada; 4 Department of Biology, McGill UniversityMontreal, Quebec, Canada; 5 MapprSan Francisco, California; 6 Santa Fe InstituteSanta Fe, New Mexico

**Keywords:** Complexity, diet, diversity, predation, sampling area, scale dependence, trophic interactions

## Abstract

This study asks whether the spatial scale of sampling alters structural properties of food webs and whether any differences are attributable to changes in species richness and connectance with scale. Understanding how different aspects of sampling effort affect ecological network structure is important for both fundamental ecological knowledge and the application of network analysis in conservation and management. Using a highly resolved food web for the marine intertidal ecosystem of the Sanak Archipelago in the Eastern Aleutian Islands, Alaska, we assess how commonly studied properties of network structure differ for 281 versions of the food web sampled at five levels of spatial scale representing six orders of magnitude in area spread across the archipelago. Species (S) and link (L) richness both increased by approximately one order of magnitude across the five spatial scales. Links per species (L/S) more than doubled, while connectance (C) decreased by approximately two-thirds. Fourteen commonly studied properties of network structure varied systematically with spatial scale of sampling, some increasing and others decreasing. While ecological network properties varied systematically with sampling extent, analyses using the niche model and a power-law scaling relationship indicate that for many properties, this apparent sensitivity is attributable to the increasing S and decreasing C of webs with increasing spatial scale. As long as effects of S and C are accounted for, areal sampling bias does not have a special impact on our understanding of many aspects of network structure. However, attention does need be paid to some properties such as the fraction of species in loops, which increases more than expected with greater spatial scales of sampling.

## Introduction

A number of ecological network studies have explicitly evaluated the impact of sampling effort, or the temporal scale of sampling, on our understanding of the structure of food webs (Winemiller 1989, 1990; Martinez 1991, 1993; Tavares-Cromar and Williams 1996; Goldwasser and Roughgarden 1997; Hawkins et al. 1997; Bersier et al. 1999; Martinez et al. 1999; Banasek-Richter et al. 2004) and more recently pollination networks (Nielsen and Bascompte 2007; Hegland et al. 2010; Tylianakis et al. 2010; Chacoff et al. 2011; Rivera-Hutinel et al. 2012). In contrast, very few studies have explicitly explored how the spatial scale of sampling affects food web network structure. To date, sampling effort in food webs has been studied indirectly by analyzing incomplete food webs or setting post hoc criteria to systematically exclude species and/or links from existing datasets (e.g., Winemiller 1990; Goldwasser and Roughgarden 1997; Martinez et al. 1999). More direct field-based manipulation of sampling effort, meanwhile, has been limited to pollination networks (e.g., Nielsen and Bascompte 2007; Hegland et al. 2010) where experiments are easier to implement. Increasingly, studies focusing on topics such as biogeography of ecological networks statistically account for potential effects of variable sampling effort underlying different datasets (e.g., Olesen and Jordano 2002; Ollerton and Cranmer 2002; Devoto et al. 2005; Trojelsgaard and Olesen 2013). Given long-standing interest in spatial aspects of food webs (Holt 2002) and increasing research on spatially explicit food web modeling (McCann et al. 2005; Amarasekare 2008; Gravel et al. 2011; Masol et al. 2011), it would be useful to have more empirical studies that document and assess the relationship between spatial scale and ecological network structure.

One early, speculative attempt to consider spatial sampling effort pointed out that fractions of top, intermediate, and basal taxa should change significantly across smaller scales and reach 0%, 95%, and 5%, respectively, at large regional to global scales (Martinez and Lawton 1995). While the study used admittedly “crude” data, focused on a few metrics, and ignored the issue of co-occurrence of taxa, the authors were thinking in the broadest possible sense about the spatial scale of food web data, represented indirectly by eight orders of magnitude of species richness. Another study developed a “link-area” model that accurately predicted how numbers of trophic links scale with area for small to large aquatic food webs, but did not consider details of network structure (Brose et al. 2004). There have been very few relevant field-based studies. One study of differences between patch- and reach-scale river food web structures reported lower connectance in the larger reach-scale webs, which the authors suggested reflected the integration of non-co-occurring species from different patch-scale webs into cumulative reach-scale webs (Thompson and Townsend 2005). Another study focused on nestedness of pollination networks by compiling network data across combinations of one, two, three, and four sites to represent increasing spatial scale. The study found that nestedness and connectance were stable across the four scales (Nielsen and Bascompte 2007).

Any assessment of the impact of spatial scale of sampling on food web structure needs to take into account systematic changes in structure with the diversity (species richness) and complexity (measured as link density or connectance) of the network (e.g., Dunne et al. 2004, 2013). In other words, food web structure is scale dependent on diversity and complexity (Riede et al. 2010). Increasing the spatial scale of sampling will generally increase the species richness represented in a food web or other ecological network as more species are encountered. It will also result in more links, which may or may not alter link density or connectance depending on the relationship of the growth of species with links. It may be the case that observed changes in particular aspects of network structure such as mean trophic level or fraction of omnivorous species across spatial scales are primarily a result of the changes in species richness, link richness, and their relationship (Dunne et al. 2004, 2013). Alternatively, there may be differences beyond what is predicted by changes in diversity and complexity, which could suggest that other factors influence observed differences in trophic organization at various spatial scales.

An improved understanding of how sampling effort affects ecological network structure is important for both fundamental ecological knowledge (Dunne 2006; Blüthgen 2010) and the application of network analysis in conservation and management (Hegland et al. 2010; Tylianakis et al. 2010). Although ecological networks ideally describe the interactions among co-occurring species (i.e., species that can encounter each other) in a habitat, it is not obvious what the relevant spatial boundaries are for most ecosystems. Even for systems like lakes or ponds where habitat delineations are fairly clear, a food web still depends on some sort of sampling or compiling of information that can and does occur at various explicit or implicit spatial scales. As with temporal sampling effort, it is important to understand whether an ecological network based on sampling a small area provides the same information about trophic organization as one based on a larger area, with similar attendant relevance for questions that range from basic to applied.

Here, we present a new, highly resolved food web for the marine intertidal ecosystem of the Sanak Archipelago in the Eastern Aleutian Islands of Alaska. We assess whether and how trophic organization varies across hundreds of versions of the intertidal food web at five spatial scales of sampling from quadrats up to locales spread across the archipelago. Our analyses and comparisons uncover some scale-dependent properties of food webs and show the importance of controlling for differences in the numbers of species and links in cross-scale comparisons of network structure.

## Materials and Methods

### Study system

**Figure 1:**
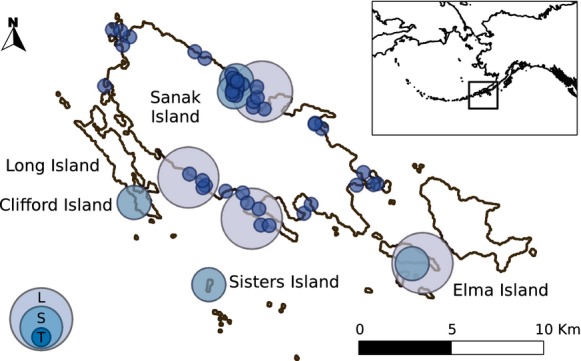
Map of the Sanak Archipelago and locations of food webs. Small circles display the locations of intertidal transects (T). Quadrats were located along the transects. Larger circles indicate the placement of sites (S) and locales (L).

The Sanak Archipelago lies in the Eastern Aleutian Islands, south of the Alaska Peninsula, in the North Pacific Ocean (Fig.1). The coastline contains a mix of semi-exposed rocky intertidal habitats interspersed with protected sedimented and boulder-strewn shores. Rocky shorelines are characterized by kelps, primarily species of *Alaria* and *Laminaria*. A general description of the archipelago, its history, and the intertidal ecosystem is provided by Maschner et al. (2009).

### Field methods

We cataloged taxa living in the intertidal zone at five spatial scales ranging from 0.25 m^2^ to 24 km^2^, representing six orders of magnitude of area. The five spatial scales are referred to as quadrats, transects, sites, locales, and the archipelago. At the smallest scale, we identified all taxa present in 0.25-m^2^ quadrats (0.5 m on a side) along 39 transects that were laid across the intertidal zones around the Sanak Islands. In June 2004, transects were spaced somewhat regularly along the coast of the main island, irrespective of habitat (Fig.1). Transects spanned the entire intertidal zone, perpendicular to the shoreline, from mean lower low water (MLLW) at chart datum to the lower edge of terrestrial biota (generally between six and 10 ft above MLLW). Wherever possible, additional sampling was conducted below MLLW. Within Pauloff Harbor, on the north side of Sanak Island, transects were placed at 300 m intervals along the shore, between the two outer edges of the harbor. Along each transect, quadrats were placed every vertical foot above MLLW. The identity of all macro-organisms present in the quadrat was recorded at the lowest taxonomic resolution possible in the field. Taxa inhabiting the undersides of rocks that were movable were also included. In quadrats where the substrate was sediment, we extracted one round core with a diameter of 10 cm, to a minimum depth of 30 cm. The sediment was then washed through a sieve with 1-mm mesh, and all remaining macro-organisms were identified to the lowest taxonomic resolution possible in the field.

The quadrat data were used to create lists of all taxa observed at two spatial scales. At the smallest scale, we created a catalog of taxa found in each of the 339 quadrats. Thirty-nine larger transect-scale catalogs were created by combining all taxa observed in the quadrats placed along each transect. The approximate area of the transect scale was 37.5 m^2^ given that the average transect was 75 m long (dependent on the slope of the shore) and 0.50 m wide.

To catalog taxa in larger areas, we conducted thorough searches of the intertidal zone at two additional spatial scales: five sites and four locales, averaging approximately 0.23 km^2^ and 0.88 km^2^, respectively. We documented as many taxa as possible by searching the intertidal zone irrespective of habitat or tidal height. All macroscopic organisms, including algae, invertebrates, and mammals, were identified to the lowest possible taxonomic resolution and recorded. We did not include taxa that live primarily outside of the intertidal zone but visit or forage there occasionally, such as birds, terrestrial mammals, and marine fish. The five 0.23-km^2^ sites (Pauloff Harbor, Clifford Island, Bird Rock, Elma Island, and Sisters Island; see Fig.1) were cataloged in June 2007. Three researchers searched the intertidal area between MLLW and the terrestrial biota for approximately 4 h each (2 h before and after low tide). In June 2006, a similar protocol was used to catalog four larger 0.88-km^2^ locales (Fig.1). The same three researchers each cataloged taxa observed during 12 h of searching (2 h before and after low tide on three separate days). Finally, a single archipelago-wide species catalog was created, representing a sampling area of 23.5 km^2^, by including all intertidal taxa observed during both the quadrat- and search-based protocols described previously. The areas of the sites, locales, and archipelago were estimated based on the assumption that the intertidal zone is 75 m wide, which was then multiplied by the length of shoreline searched in the case of sites and locales, or by the total length of the shoreline of the five islands for the archipelago-wide area. Shoreline lengths were derived from the Global Self-consistent, Hierarchical, High-resolution Geography Database (Wessel and Smith 1996).

### Food webs

A cumulative, archipelago-wide food web was constructed for all observed taxa containing feeding links observed directly by the authors in the field or described in peer-reviewed publications, dissertations, or technical reports (based on 6712 feeding observations collected from 745 sources). The sources used a variety of methods to assess whether predation occurred, including direct observation of feeding in the laboratory or field, gut or scat contents, and expert opinion. This approach to compiling feeding links was employed by Martinez (1991) and many subsequent studies. The food web integrated feeding links for all life stages and anatomical structures of consumers and resources to create a single set of links for each taxon (Dunne et al. 2013).

We strived for the highest possible taxonomic resolution in both feeding data and species identifications in the field. When information about consumers or resources was incomplete, or species could not be identified in the field, we combined taxa into coarser groups according to the taxonomy provided by the Integrated Taxonomic Information System (ITIS 2010). Thus, the food web is comprised of taxa from a variety of taxonomic levels from species up through phyla. The decision of whether or not to lump taxa into larger groups was made using expert knowledge about the natural history of the system and taxon's expected resources. Generally, large, distinct, or well-studied taxa were included in the food web at the species level, while taxa that were smaller and more difficult to identify were combined into families. Some taxa, such as diatoms, could not be distinguished in the field and were therefore included at even higher taxonomic levels (e.g., division Bacillariophyta). When aggregating taxa, all links to lower-level taxa were included as links for the aggregated taxon. Taxa that lacked names in the traditional Linnaean taxonomic hierarchy were included in the web as “morphospecies.” For example, sea urchin and sand dollar are morphospecies that do not fall into Linnaean subgroups within class Echinoidea. Synonym names reported by publications were converted into currently accepted taxonomic names according to the ITIS.

The archipelago-wide food web was used to construct spatially localized food web datasets for places based on their species catalogs. In creating the archipelago food web as well as various food webs at smaller spatial scales, we assumed that if two species have a feeding link in one place, they are linked everywhere they co-occur. This assumption has been used in other studies featuring multiple web instances for a particular habitat or habitat type (e.g., Havens 1992; Piechnik et al. 2008; Baiser et al. 2011) and has the potential to overestimate the number of links per species in any one place. Taxa that occurred in a sample but were not linked to any other taxa within that unit were dropped from that food web. Then, Bacillariophyta, Bacteria, biofilms, Cyanophycota, detritus, phytoplankton, and zooplankton were assumed to be present in every remaining food web. A total of 388 food webs were compiled (339 quadrat webs, 39 transect webs, five site webs, four locale webs, and one archipelago-wide web, provided in Appendix S1).

**Figure 2:**
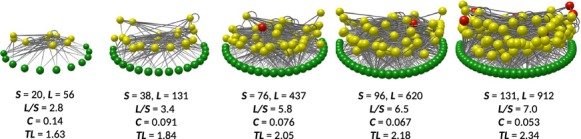
Diagrams of example food web networks from each of the five spatial scales studied (quadrat, transect, site, locale, and archipelago, from left to right). Spheres represent taxa and lines between them represent directional feeding links. Primary producers, invertebrates, and vertebrates are shown in green, yellow, and red, respectively. The vertical axis indicates short-weighted trophic level.

Prior to analyses, any taxa with the same predators and prey were aggregated into single trophic species (Briand and Cohen 1984), a convention for comparative food web structure analysis (e.g., Dunne et al. 2013). We excluded the 104 quadrat-scale and three transect-scale food webs that had fewer than 10 trophic species because simulations using the niche model (Williams and Martinez 2000) and random models for intertidal webs ranging from two to 35 trophic species showed that models produce systematically large errors below 10 taxa (unpublished data). Thus, in our analyses, we included 235 quadrat webs, 36 transect webs, five site webs, four locale webs, and one archipelago web, for a total of 281 intertidal food webs. An example food web from each scale is shown in Figure2.

### Analyses

The structure of each food web was characterized in two ways. First, we calculated four fundamental properties: the number of species (S), the number of links (L), the mean links per species (L/S), and connectance (C), which measures the proportion of all possible links realized (in this case, directed connectance = L/S^2^). Second, we calculated 14 commonly studied properties of network structure: the fractions of top, intermediate, and basal species (Top, Int, and Bas); the fractions of cannibals, herbivores, omnivores, and species in loops (Can, Herb, Omn, and Loop); the standard deviations of normalized total links, generality, and vulnerability (LinkSD, GenSD, and VulSD); the mean short-weighted trophic level of all species (TL); the mean maximum trophic similarity of species (MaxSim); the mean shortest number of links between species pairs (Path); and the mean clustering coefficient (Clust) (see Table 1 in Dunne et al. 2013 for definitions).

The same set of properties was also computed for ten other food webs from a variety of habitats, including three marine systems (Benguela, Caribbean Reef, and N.E. U.S. Shelf), two estuaries (Chesapeake Bay and St. Marks Estuary), three lakes or ponds (Bridgebrook Lake, Skipwith Pond, and Little Rock Lake), and two terrestrial ecosystems (Coachella Valley and St. Martin Island) with trophic species richness of 25 to 92 (see details in Williams and Martinez 2008). These 10 food webs, which have been a part of many previous studies of ecological network structure (e.g., Williams and Martinez 2000, 2008; Dunne et al. 2002, 2004; Stouffer et al. 2005, 2007; Allesina and Pascual 2009; Staniczenko et al. 2010), are not meant to be comprehensive, but rather they are provided as a reference for how the intertidal webs compare to some commonly studied webs with a similar range of species richness.

As the 14 network structure properties covary with the diversity and complexity of the food web (Vermaat et al. 2009), and as S and L vary with spatial scale, potentially impacting L/S and C, we used two separate methods to account for expected differences in the raw values of the properties with varying S and C. First, we modeled a power-law scaling relationship between the 14 network properties (P) and S and C (Riede et al. 2010) in a general linear model (P ∼ log_10_(S) + log_10_(C)) with a binomial distribution. Then, for each property of each food web, we measured residual variation (RV) from the best-fit regression line (as in Digel et al. 2014). Negative and positive power-law RV values indicate model under- and overestimation, respectively. Second, we used the niche model (Williams and Martinez 2000), to generate 1000 niche model webs with the same S and C as each of the 281 intertidal webs and the 10 additional food webs. For each property of each web, we calculated model error (ME): the normalized difference between the niche model's median value and the empirical value (Williams and Martinez 2008). ME > ¦1¦ indicates that the empirical property falls outside 95% of the modeled values. The MEs are conversely related to power-law RVs, with negative and positive MEs indicating model underestimation and overestimation of the empirical value, respectively.

We used Tukey boxplots to compare the raw values of each property across the set of webs at the ordinal quadrat, transect, site, and locale scales (the archipelago scale has a single value per property), as well as the 10 published webs, and did the same for MEs for each property. The boxplots show the mean, interquartile range (IQR), maximum datum within 1.5 IQR of the box (whiskers), and data lying outside the whiskers. Comparing the boxplots of the raw property values to power-law model RV and niche model ME values allows assessment of the degree to which any trends in the values within and across scales are accounted for by the S and C normalization provided by the two models.

## Results

### Fundamental properties

**Figure 3:**
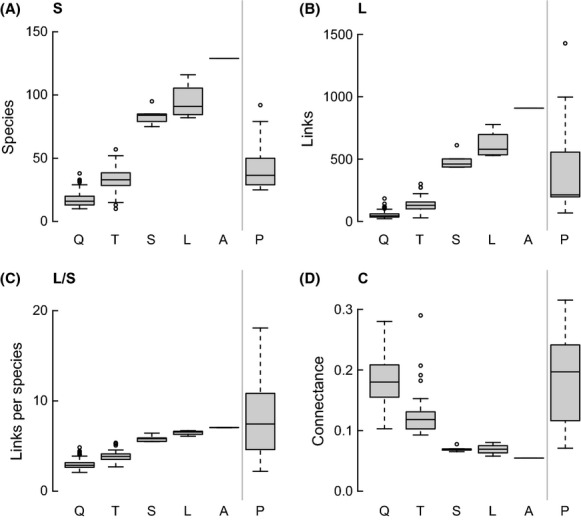
The number of taxa (A), links (B), links per taxon (C), and connectance (D) of quadrats (Q), transects (T), sites (S), locales (L), archipelago-wide data (A), and the 10 previously published food webs (P).

The archipelago-wide food web has 131 taxa and 912 links prior to trophic species aggregation. All further results are for trophic species versions of the various webs. The S, L, and L/S of the intertidal food webs increase systematically across the gradient in spatial resolution (Fig.3). At the smallest scale, quadrat-scale food webs have, on average, 17 trophic species, 55 links, and 2.9 L/S. These numbers increase to 129 trophic species, 909 links, and 7.0 L/S for the largest scale archipelago food web. Connectance decreases with increasing spatial scale, from a mean of 0.18 in quadrat-scale food webs to 0.06 for the archipelago food web (Fig.3). Thus, over the five spatial scales and six orders of magnitude in area, S and L both increased by approximately an order of magnitude, L/S more than doubled, and C decreased by approximately two-thirds.

### Network structure

**Figure 4:**
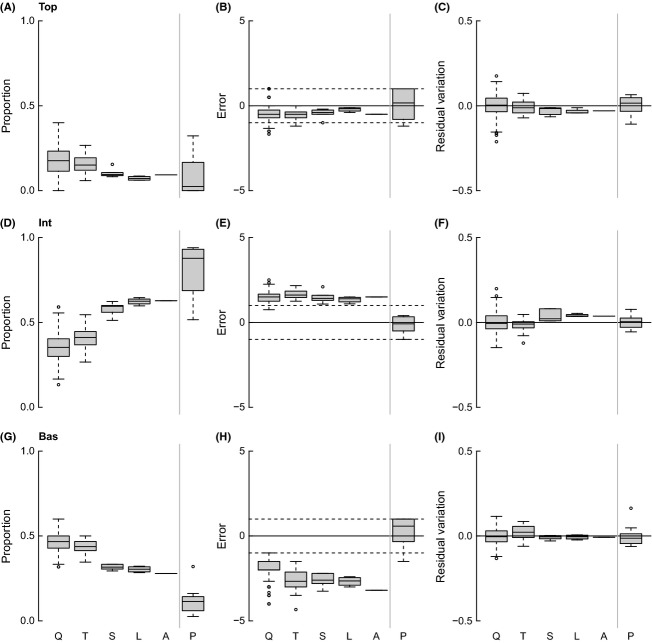
Plots of raw values (left column), deviations from estimates calculated by the niche model (center column), and residual variation (RV) from a power-law scaling model (right column) for proportions of top (A–C), intermediate (D–F), and basal (G–I) taxa across spatial scales (as abbreviated in Fig.3). Negative niche model errors indicate that the niche model is underestimating the value for a given food web property; positive values indicate that the niche model is overestimating the property. Negative and positive power-law RVs indicate that the power-law model is overestimating and underestimating the property, respectively.

**Figure 5:**
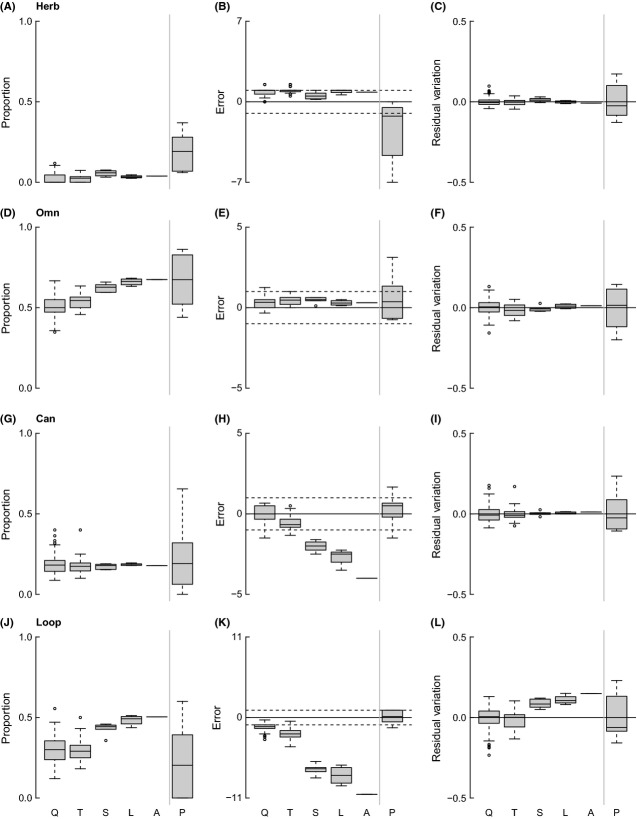
Plots of raw values (left column), deviations from estimates calculated by the niche model (center column), and residual variation from a power-law scaling model (right column) for proportion of herbivorous (A–C), omnivorous (D–F), and cannibalistic (G–I) taxa, and the fraction of taxa in loops (J–L) across spatial scales (as abbreviated in Fig.3).

Top and Bas decrease with increasing spatial scale, with a coinciding increase in Int (Fig.4). However, the niche ME and power-law RV for these properties are generally similar across scales, suggesting that the observed trends in Top, Int, and Bas with sampling area are primarily a function of changes in S and C. Herb and Can are relatively constant over the five spatial scales, while Omn and Loop increase with spatial scale (Fig.5). Niche and power-law model results suggest that variation in Herb and Omn, which show stable ME and RV across spatial scales, are accounted for by changes in S and C. Niche model MEs for Can and Loop strongly decrease with increasing sampling. The power-law RV of Can does not vary, while Loop increases concomitantly with the decrease in Loop niche ME.

**Figure 6:**
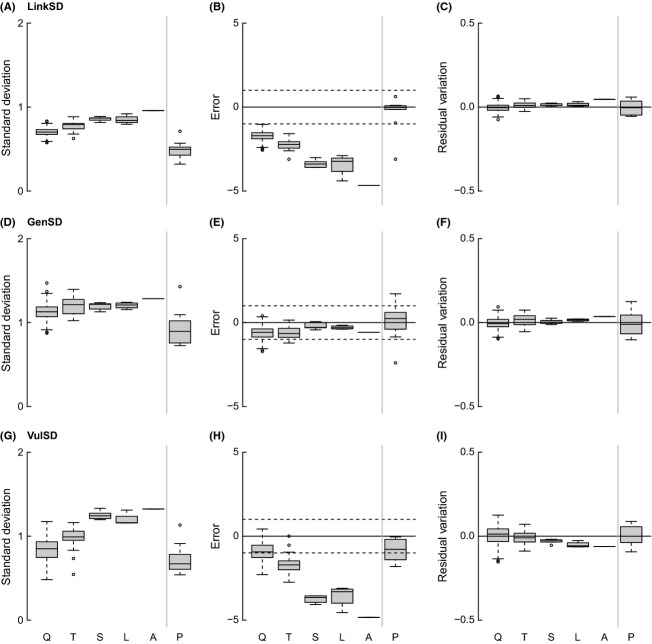
Plots of raw values (left column), deviations from estimates calculated by the niche model (center column), and residual variation from a power-law scaling model (right column) of standard deviations of normalized total links (A–C), generality (D–F), and vulnerability (G–I) across spatial scales (as abbreviated in Fig.3).

**Figure 7:**
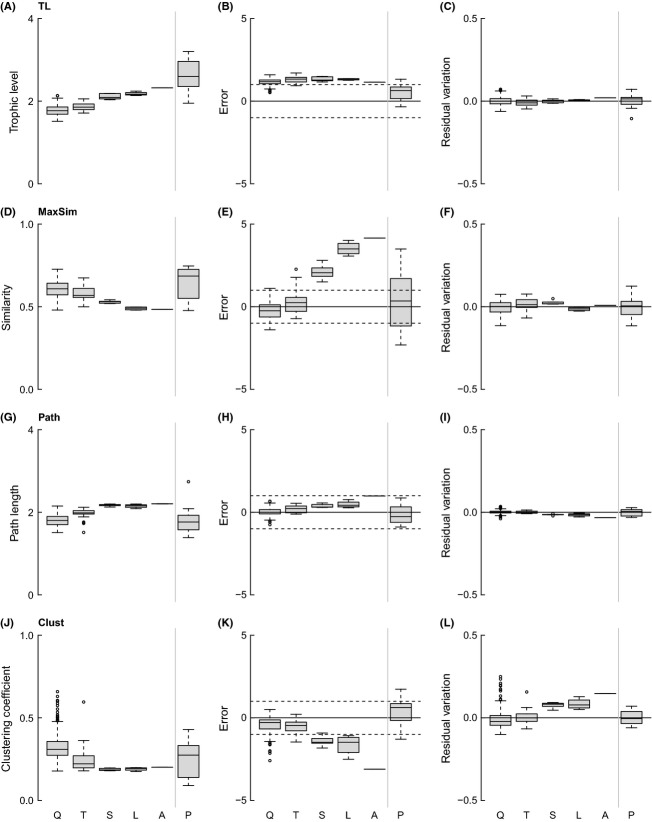
Plots of raw values (left column), deviations from estimates calculated by the niche model (center column), and residual variation from a power-law scaling model (right column) of the mean short-weighted trophic level of all species (A–C), maximum trophic similarity of species (D–F), shortest number of links between species pairs (G–I), and clustering coefficient (J–L) across spatial scales (as abbreviated in Fig.3).

GenSD and LinkSD increase slightly across spatial scales, while VulSD increases strongly with scale (Fig.6). Niche model results suggest that variation in GenSD is accounted for by changes in S and C, given the stability in MEs across scales, while MEs for VulSD and LinkSD strongly decrease with spatial scale. The power-law relationship of GenSD, LinkSD, and VulSD is constant across spatial scales. TL and Path increase slightly over spatial scales, MaxSim is relatively stable across scales, and Clust decreases slightly with spatial scale (Fig.7). Niche model results suggest that changes in TL and Path are accounted for by changes in S and C, given the stability in MEs across scales, while model MEs increase strongly for MaxSim and decrease for Clust with spatial scale. Power-law results agree with those of the niche model except for MaxSim, which is consistently near zero across all spatial scales.

### The fit of the niche model

Niche MEs fall between 1 and −1 for Top, Herb, Omn, GenSD, and Path across all spatial scales, indicating good fit of the model to the data. The niche model consistently slightly overestimates Int and TL regardless of spatial scale. The niche model increasingly underestimates Bas, Can, Loop, VulSD, LinkSD, and Clust, and increasingly overestimates MaxSim, with increasing spatial scale. At the smallest spatial scale sampled, there is good fit of the niche model displayed for Top, Herb, Omni, Can, GenSD, VulSD, MaxSim, Path, and Clust (Figs.7).

### Comparison to other webs

The 10 other webs examined have mean S, L, L/S, and C that fall between the mean values for the transect-scale and site-scale intertidal food webs. Unsurprisingly, these 10 webs, which represent a variety of habitats, show greater variability in their fundamental properties compared to any set of intertidal webs at a particular scale (Fig.3). To compare structural properties, we focus on niche and power-law MEs, as they provide the most direct comparison (Figs.7). For four properties, Top, Omn, GenSD, and Path, the variability of ME and RV across the 10 webs encompasses or is comparable to the variability in modeled error of the intertidal networks across scales. However, in most cases, the ME and RV of the intertidal webs at all scales are quite different from the modeled error across the 10 webs. The niche model tends to fit the 10 webs more closely, in terms of mean ME, than the intertidal webs (particularly compared to results for larger spatial scales) for nine of 14 properties: Int, Bas, Can, Loop, LinkSD, VulSD, TL, MaxSim, and Clust. For Herb, the niche model tends to slightly overestimate it for the intertidal webs regardless of scale and underestimate it, with high variability, for the 10 other webs.

## Discussion

For decades, ecologists have debated whether structural properties of food webs vary across various scales of measurement. Scale can refer to the spatial, temporal, or statistical domain of the data, where statistical domain refers to variation in properties such as diversity or complexity (Martinez 1994; Martinez and Dunne 1998). Most previous ecological network studies have focused on either the statistical domain (i.e., relationship of structure to S and C) or the temporal domain (i.e., relationship of structure to sampling effort). Here, we help extend the discussion to the spatial domain by presenting an empirical assessment of whether commonly studied properties of network structure differ across five ordinal levels of spatial scale representing six orders of magnitude in area. This study extends beyond two relevant, prior empirical studies (Thompson and Townsend 2005; Nielsen and Bascompte 2007) by looking at more spatial scales over greater orders of magnitude of change, by examining more aspects of network structure, and by identifying variation in structure attributable to changes in species and link richness at different spatial scales.

In the current analysis of intertidal food web structure at five spatial scales from quadrat to archipelago, ranging six orders of magnitude of area from 0.25 m^2^ to 24 km^2^, species richness (S) and link richness (L) increased with spatial scale, as expected. S increased by slightly more than an order of magnitude, while L increased by slightly less than an order of magnitude. Links per species (L/S) also increased with scale, more than doubling on average, while connectance (C = L/S^2^) decreased with scale, with an approximately two-thirds reduction. While one prior study also observed a decrease in connectance from patch- to reach-size stream food webs (Thompson and Townsend 2005), they hypothesized that it was due to the artificial lumping of species in heterogeneous patch-size webs (representing different types of stream habitat) into larger reach-size webs, resulting in false co-occurrence of taxa that never interact. While that may have been the case in that study, it does not account for the decrease in this study, where all the species co-occur and potentially interact at all of the spatial scales. The other study, which focused on connectance in pollination networks at four spatial scales of sampling, found connectance to be stable (Nielsen and Bascompte 2007). The different result compared to the current study could be due to the difference between sampling a full set of species versus just plants and pollinators, or due to the more limited range of effective areas being sampled in the pollination network study.

Beyond the fundamental properties reflecting the basic diversity and complexity of food webs, of 14 commonly measured structural properties of networks, all but three (Herb, Can, and MaxSim) varied systematically with spatial scale. In most cases, the properties that varied increased with spatial scale. In the case of fractions of top, intermediate, and basal taxa, Top and Bas decreased with spatial scale, which necessarily meant that Int increased with spatial scale, as generally predicted by an earlier, speculative study encompassing a much larger range of spatial scales (Martinez and Lawton 1995). Based on these results, it would be easy to conclude that most aspects of network structure do indeed vary, and vary systematically, with spatial scale of sampling. Our results show that they obviously do. However, given prior work showing that network structure varies systematically with changes in fundamental properties S, L, L/S, and C (Williams and Martinez 2000; Dunne et al. 2002; Williams et al. 2002; Dunne et al. 2004; Stouffer et al. 2005, 2007; Williams and Martinez 2008; Vermaat et al. 2009; Riede et al. 2010; Dunne et al. 2013), such variation with scale of spatial sampling may be largely accounted for by changes in structure driven by these fundamental properties.

We normalized for the effect of changes in fundamental properties on network structure using the niche model, the best-available model of food web structure, and a power-law scaling model (Williams and Martinez 2000, 2008; Riede et al. 2010). This model-based comparative approach allows us to understand which trends in structure with spatial scale are primarily due to changes in the diversity and complexity of food webs at different spatial scales, and which trends in structure are unexpected. This type of approach has been used previously to explore related questions about whether food web network structure varies across habitats, through deep time, and when parasites are included versus excluded in datasets (e.g., Dunne et al. 2004, 2008, 2013, 2014; Digel et al. 2014). The finding that a power-law model can be reasonably well fit to the various network properties reinforces the conclusion that these attributes do in fact vary consistently with S and C. The niche model analyses provide additional insights by identifying spatial variation in properties that were different from those found in the niche model and were not highlighted by the power-law scaling model.

The niche model analysis indicated that eight of the 14 studied network structure properties have trends across spatial scales that appear to be primarily accounted for by changes in diversity and complexity with spatial scale, as indicated by stable niche MEs across spatial scales. Thus, Top, Int, Bas, Herb, Omn, GenSD, TL, and Path exhibited trends that are largely predicted by the variation found in niche model networks with the same increasing species richness (S) and decreasing connectance (C) found in the empirical webs with increasing spatial scale of sampling. However, five properties (Can, Loop, LinkSD, VulSD, and Clust) displayed strong decreases in MEs with spatial scale. The increasingly negative MEs indicate that the niche model increasingly underestimated those properties at larger spatial scales of sampling. One property displayed positive and increasing MEs, indicating increasing overestimation by the niche model with spatial scale (MaxSim).

The fact that six properties in question showed systematic increases (or decrease for MaxSim) of niche MEs with area sampled, rather than some more noisy change, suggests that the additional effects beyond increasing S and decreasing C are linked to a systematic impact of the sampling of species and their links with area, as opposed to some other effect. The fraction of cannibalistic species (Can), for example, is well predicted by the niche model at the smallest spatial scale (quadrat), but as sampling area increases to the full archipelago, there is an increasing “overabundance” of cannibalistic species relative to niche model predictions for webs with increasing S and decreasing C. Some effect beyond the simple impacts of increasing diversity and complexity on niche model structure is at play. Williams and Martinez (2008) observed that the ME of the niche model predictions of Can and Loop was close to zero across 10 networks ranging from 25 to 92 nodes and up to 997 links. In this case, however, cannibalistic species appear more likely to be sampled or observed at larger spatial scales and thus end up representing increasing fractions of the full set of sampled species.

Ecological mechanisms may underlie the systematic MEs, such as the trend for the niche model to increasingly underestimate VulSD in larger food web networks. Contrary to the predictions of the niche model, raw VulSD increases with scale (Fig.6g–h). This greater variability in vulnerability is caused by a subset of prey species forming hubs that contain many more incoming links than the average node. In the smallest food webs, the vulnerability of all types of prey is consistently low. In larger networks, however, aggregated prey groups such as detritus, diatoms, phytoplankton, and zooplankton have disproportionately greater numbers of predators relative to other taxa. The same is true for a variety of highly resolved prey including seaweeds (*Fucus* sp. and Ulvaceae), snails (*Littorina* sp.), a mussel (*Mytilus* sp.), and the urchin *Strongylocentrotus droebachiensis*. The observation that these prey taxa form preferential attachment points for predators may indicate that they also serve important functional roles in these intertidal communities.

Our results also provide a new empirical assessment of the niche model, which has displayed generally good fit to food webs with S < ∼100 (Williams and Martinez 2000, 2008; Dunne et al. 2004; Stouffer et al. 2005, 2007) but increasingly poor fit for larger food webs (Williams and Martinez 2008; Dunne et al. 2013, 2014). At the two smallest spatial scales of sampling, quadrat and transect (S = 17 and S = 33, respectively), only five of 14 properties (Int, Bas, Loop, LinkSD, and TL) display poor fit with MEs > ¦1¦, with TL only slightly overestimated by the niche model. However, at the larger spatial scales of site and locale (S = 84 and S = 95), as well as the single archipelago food web (S = 129), an additional four properties display poor fit (Omn, VulSD, MaxSim, and Clust). Thus, the more species-rich intertidal food webs at larger spatial scales have only five of 14 properties with good fit to the niche model, while the ten other food webs examined (Williams and Martinez 2008) have mean MEs that display good fit for 13 of 14 properties.

The differences in niche model fit between the new intertidal webs and 10 previous webs, as well as the reasonably good fit of the niche model to intertidal webs at the two smallest spatial scales of sampling, make sense when we consider that the mean S and L of the 10 other webs are 45 and 424, respectively, which are most similar to the mean values of 33 and 129, respectively, for the intertidal transect food webs (Fig.3), the second smallest spatial scale of sampling investigated. However, even at the smallest spatial scales of sampling, the intertidal food webs are still more poorly fit by the niche model than the 10 other webs. This is likely related in part to the higher resolution of basal taxa in the intertidal webs compared to other webs. The niche model significantly underestimates Bas for the intertidal webs at all spatial scales, while it slightly overestimates Bas for the other webs (Fig.4). In other words, the niche model generates structure that predicts proportions of basal taxa that are similar to what is seen in datasets that have lower resolution of basal taxa.

In summary, this methodological study indicates that when changes in S and C are accounted for, the spatial scale of sampling, in this case from 0.25 m^2^ to 24 km^2^, does not bias our understanding of some aspects of food web network structure. Thus, while food webs compiled with data drawn from larger spatial areas will represent greater biodiversity of the system, as well as potential changes to complexity measures such as L/S and C (in this case, increased L/S and reduced C), many aspects of network structure are robust to the changes in spatial scale of sampling. However, our study also indicates that attention does need to be paid to some properties such as fraction of looping and cannibalistic species (Loop and Can) which increase more than expected given changes in diversity and complexity at greater spatial scales of sampling. Structural properties that are related to spatial scale appear to be sensitive in systematic ways. These trends are apparent when looking at just a few spatial scales of sampling – here, at the three smallest scales of sampling, from quadrat (0.25 m^2^) to site (0.23 km^2^). In future studies, it would be useful to understand the generality of these results based on similar studies in other intertidal habitats and well as other kinds of ecosystems.

## Data Accessibility

The 281 intertidal food webs analyzed here are available in Appendix S1 and available from the Dryad Digital Repository: http://dx.doi.org/10.5061/dryad.g1qr6.

## Supporting Information

Appendix S1. The food web data compiled for this study, consisting of 339 quadrats, 39 transects, five sites, four locales, and one archipelago-wide network. The table provides a unique numeric identifier per food web (*WebID*), the spatial scale (*WebScale*; distinguishing quadrats [Q], transects [T], sites [S], locales [L], and the archipelago-scale [A]), a sample number (*WebUnit;* to identify individual transects [quadrat- and transect-scale samples], sites, or locales), and the Integrated Taxonomic Information System's (ITIS) Taxonomic Serial Number (TSN) and name of each predator and prey (*PredTSN*, *PreyTSN*, *PredName*, and *PreyName*, respectively). Taxa lacking entries in ITIS have a unique custom TSN prepended with “san”. Data are also available from the Dryad Digital Repository (http://dx.doi.org/10.5061/dryad.g1qr6).
